# MTOR-Mediated Autophagy Is Involved in the Protective Effect of Ketamine on Allergic Airway Inflammation

**DOI:** 10.1155/2019/5879714

**Published:** 2019-01-09

**Authors:** Hongyun Zou, Li-Xia Wang, Muzi Wang, Cheng Cheng, Shuai Li, Qiying Shen, Lei Fang, Rongyu Liu

**Affiliations:** ^1^Department of Pulmonary, Anhui Geriatric Institute, The First Affiliated Hospital of Anhui Medical University, Jixi Road 218, Hefei, Anhui 230022, China; ^2^Department of Anesthesiology, The First Affiliated Hospital of Anhui Medical University, Jixi Road 218, Hefei, Anhui 230022, China

## Abstract

Unresolved inflammation underpins the pathogenesis of allergic airway diseases, such as asthma. Ketamine, accepted as a promising therapy for resistant asthma, has been demonstrated to attenuate allergic airway inflammation. However, the anti-inflammatory mechanism by ketamine in this setting is largely unknown. We aimed to investigate whether autophagy was involved in the protective effect of ketamine on allergic airway inflammation. Female C57BL/6 mice were sensitized to ovalbumin (OVA) and treated with ketamine at 25, 50, or 100 mg/kg prior to OVA challenge. In this model, the pulmonary morphological findings and airway inflammation were significantly inhibited at 50 mg/kg but not at 25 or 100 mg/kg. Moreover, 50 mg/kg ketamine abrogated the increased concentrations of inflammatory cytokines in bronchoalveolar lavage fluid (BALF) of allergic mice, as well as activated the expression of phosphorylated mammalian target of rapamycin (p-MTOR) and inhibited autophagy in allergic mice. To confirm whether the effect of 50 mg/kg ketamine on asthma was mediated by inhibiting autophagy, rapamycin was administered to mice sensitized to OVA and exposed to 50 mg/kg ketamine. All of the effect of 50 mg/kg ketamine was reversed by rapamycin treatment, including increased p-MTOR and decreased autophagy. Taken together, the present study demonstrates that 50 mg/kg ketamine inhibits allergic airway inflammation by suppressed autophagy, and this effect is mediated by the activation of MTOR in the lungs of allergic mice.

## 1. Introduction

Allergic airway inflammation is a chronic inflammatory disease that is mediated by both innate and adaptive immunity [1]. Glucocorticoids are the most widely used anti-inflammatory drugs for allergic airway inflammation. However, 1–2% of asthma patients are entirely corticosteroid-insensitive [2]. Therefore, exploring the mechanisms responsible for this refraction and identification of alternative anti-inflammatory therapy is important.

Mammalian target of rapamycin (MTOR) is a kinase that responds to activation in both innate and adaptive immune systems. Inhibition of MTOR by rapamycin in patients has been reported to induce various inflammatory disorders, such as interstitial pneumonitis, anemia of the chronic inflammatory type, and severe forms of glomerulonephritis [3, 4]. Previous studies have shown the important role of MTOR in immunity by limiting proinflammatory mediators [5]. In human monocytes and myeloid DCs, inhibiting the MTOR signaling pathway by rapamycin increased the generation of IL-12, IL-23, tumor necrosis factor-*α* (TNF-*α*), and IL-6, as well as decreased the levels of IL-10, an anti-inflammatory cytokine [6–8]. Furthermore, a study with asthmatic mice showed that MTOR knockout significantly induced airway inflammation and increased inflammatory cells and inflammatory cytokines, such as IL-6 and TNF-*α*, in BALF [9].

MTOR also plays a critical role in regulating autophagy, which is a highly and evolutionarily conserved cellular process in various cell types, to maintain cell survival by eliminating dysfunctional organelles or proteins. In the past decade, autophagy has been demonstrated as essentially associated with inflammatory responses of lungs to infection or stresses [10]. Furthermore, emerging studies recently revealed that autophagy is importantly involved in asthma [11].

Previous reports have demonstrated the protective effects of ketamine on allergic airway inflammation diseases, such as asthma [12]. However, other studies showed no benefits of ketamine over conventional therapy for moderate-to-severe asthma [13]. Although the conflicting results exist, ketamine is suggested to be a second therapy in patients with asthma insensitive to standard therapy, especially children [13, 14]. The beneficial effects on allergic inflammation by ketamine are reported to be associated with the anti-inflammation in airways triggered by allergens [15]. Additionally, ketamine has been revealed to activate MTOR and subsequently suppress autophagy, leading to amelioration of inflammation in ischemia/reperfusion in brains [16]. However, the mechanism by which ketamine affects the allergic airway remains obscure. In the present study, we aimed to evaluate the protective effect of ketamine on allergic airway inflammation and to assess whether autophagy plays a role in this course of events.

## 2. Materials and Methods

### 2.1. Animals

Female C57BL/6 mice at 18–22 g were purchased from Shanghai Laboratory Animal Center. All of the mice were 6–8 weeks old and housed in a specific pathogen-free animal facility with a 12 h light/dark cycle. Free access to food and water was given to the mice, and all of the protocols used in this study were approved by the Committee on the Ethics of Animal Care and Use of Anhui Medical University.

### 2.2. Protocol and Treatments

**Figure 1:**
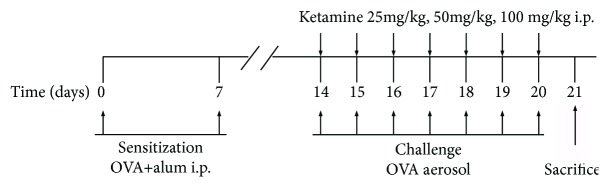
Individual mice were sensitized by intraperitoneal injection with ovalbumin (OVA) or saline on days 0 and 7, then challenged for 30 min per day with 1% aerosolized OVA or saline on days 14–21. One hour before aerosol OVA challenge, mice were injected intraperitoneally with 25, 50, and 100 mg/kg ketamine or equivalent saline.

The mice were randomly divided into five groups (*n* = 6 per group): control group (saline), OVA-induced asthma group (OVA), OVA + 25 mg/kg ketamine group (OVA+Ket25), OVA + 50 mg/kg ketamine group (OVA+Ket50), and OVA + 100 mg/kg ketamine group (OVA+Ket100) (ketamine hydrochloride, Hengrui Inc., Nanjing, China). The experimental protocol is illustrated in Figure 1. The mice were sensitized by intraperitoneal injection of 10 *μ*g OVA (Sigma, St. Louis, MO, USA) complexed with 1 mg potassium aluminium sulfate (Sangon Biotech, Shanghai, China) in 0.5 mL of saline on days 0 and 7, then challenged for 30 min per day with 1% aerosolized OVA on days 14–21. Control mice were saline-sensitized and challenged with nebulized saline solution. One hour before allergen challenge, three groups of mice (OVA+Ket25/Ket50/Ket100) were injected intraperitoneally with 25, 50, or 100 mg/kg Ket. About 24 h after the last treatment, all of the mice were killed and the lungs were collected for bronchoalveolar lavage fluid (BALF) collection. The right lungs were stored in 10% neutral formalin for histological examination, while the rest were stored at −80°C until analysis, except for samples (1–2 mm^3^) used for electron microscopy, which were fixed in 2.5% precooled glutaraldehyde.

**Figure 2:**
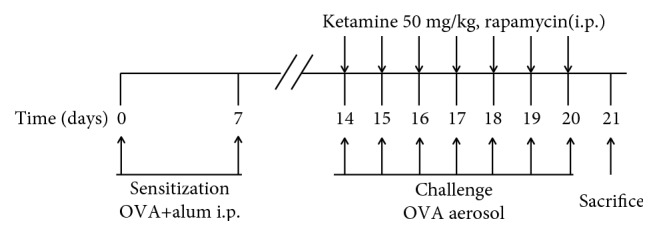
Study protocol to the administration of rapamycin and ketamine to mice sensitized to OVA.

To observe the role of MTOR activation in the effect of ketamine on allergic airway inflammation, rapamycin was administered to mice as shown in Figure 2. The mice were sensitized to OVA and treated with 50 mg/kg ketamine as indicated above. Rapamycin (Sigma, St. Louis, MO, USA) was prepared in saline containing 20% DMSO and was intraperitoneally applied 30 min prior to each administration of ketamine. The samples were collected as above about 24 h after the last treatment.

### 2.3. BALF and Differential Cell Counts

Immediately after the mice were killed, BALF was collected thrice by lavaging the left lung with 0.5 mL of ice-cold PBS. The collected BALF were centrifuged at 700*g* at 4°C for 5 min, and the cell pellets harvested from BALF were resuspended in 200 *μ*L PBS. With the cell suspension, total cell count was determined using a hemocytometer. Subsequently, Wright staining was performed to count different cell types, including neutrophils, monocytes, eosinophils, and lymphocytes under a light microscope. Cell counts were determined by a technician who was blinded to the groups.

### 2.4. Enzyme-Linked Immunosorbent Assay (ELISA)

The released cytokines, including IL-6, IL-13, IL-10, and TNF-*α*, in BALF supernatant were measured by ELISA using specific kits from Cusabio (Wuhan, China). The cut-offs for IL-6, IL-13, TNF-*α*, and IL-10 were 1.56 pg/mL, 31.25 pg/mL, 3.9 pg/mL, and 3.12 pg/mL, respectively. OVA-specific IgE was determined with ELISA kits from Cusabio (Wuhan, China). All of the levels were determined according to the manufacturer's instructions. In brief, samples or standards were pipetted into wells and incubated. After washing, detection antibodies were added and incubated. Through horseradish peroxidase (HRP) conjugate and following substrate solution incubation, the immune activity was visualized. After stopping the reaction, the values at 450 nm were measured using a microplate reader (TECAN GENios, Austria).

### 2.5. Histological Examination and Immunohistochemistry

Lung tissues fixed in formaldehyde were dehydrated by a series of ethanol solutions with increasing concentrations and then embedded in paraffin. Sections of 5 *μ*m thickness were prepared and dewaxed. After hydration, tissues were stained with hematoxylin and eosin (H&E) or periodic acid-Schiff (PAS). The observation of airway inflammation and goblet cell hyperplasia and mucus production was conducted as previously described [11]. In brief, peribronchial inflammation in airways was evaluated with an 8-point semiquantitative scoring system, while goblet cell hyperplasia was determined with Pierre Camateros' method by counting PAS-positive cells and standardized by dividing the perimeter of the basement membrane.

Immunohistochemistry for p-MTOR (Cell Signaling, Danvers, MA, USA) was performed on the hydrated sections. After antigen retrieval with citrate buffer (pH 6.0) by heating the slides for 15 min in a microwave oven, normal goat serum was applied for 30 min and then sections were incubated overnight at 4°C with the primary antibodies. A three-step technique (labelled streptavidin-biotin complex, Dako, Glostrup, Denmark) was used for visualization, and diaminobenzidine (DAB) used as a chromogen. Finally, the sections were counterstained with hematoxylin.

### 2.6. Western Blot Analysis

Lung tissue specimens were lysed with RIPA containing a protease inhibitor, and then protein concentrations were measured using a BCA Protein Assay Kit (Pierce, Rockford, IL, USA). Equal amounts of proteins were loaded into wells and electrophoresed on 10% of sodium dodecyl sulfate- (SDS-) polyacrylamide gels. Subsequently, the proteins in gels were transferred to polyvinylidene difluoride (PVDF) membranes (Millipore, Billerica, MA, USA). After blocking with nonfat milk, the membranes were probed with primary antibodies against MTOR, p-MTOR, LC3-I, LC3-II, and GAPDH (Cell Signaling, Danvers, MA, USA) and Beclin-1 (Abcam, Boston, MA, USA). After washing, the membranes were incubated with secondary antibodies conjugated with horseradish peroxidase (HRP). Immunoreactions were visualized with an ECL detection system (GE Healthcare, USA), and the intensities of protein bands were analyzed by ImageJ 1.38× software (National Institutes of Health, Bethesda, MD, USA).

### 2.7. Transmission Electron Microscopy

The lung specimens fixed in glutaraldehyde overnight were washed with cacodylate buffer (pH 7.2) three times and postfixed in 1% osmium tetraoxide. Then, samples were dehydrated in ascending concentrations of ethanol and embedded in epoxy resin. Sections of 1 *μ*m thickness were prepared and stained with toluidine blue for observation under light microscopy. Areas suitable for examination under electron microscopy were determined. Ultrathin sections (60–70 nm) were prepared and stained with uranyl acetate and lead citrate. Imaging was conducted with a transmission electron microscope (EM208S; Olympus, Tokyo, Japan). The number of vacuoles and the ratio to the cross-sectional area occupied on the sections were quantified using MetaMorph 6.1 software.

### 2.8. Statistical Analysis

GraphPad Prism 6.0 software was used for statistical analysis. All of the data were presented as the mean ± SD. Student's *t*-test was used to identify statistical significance between two groups. A one-way analysis of variance (ANOVA) followed by Tukey's tests was used to compare multiple groups. A value of *P* < 0.05 was considered statistically significant.

## 3. Results

### 3.1. Roles of Ketamine in Allergic Airway Inflammation

**Figure 3:**
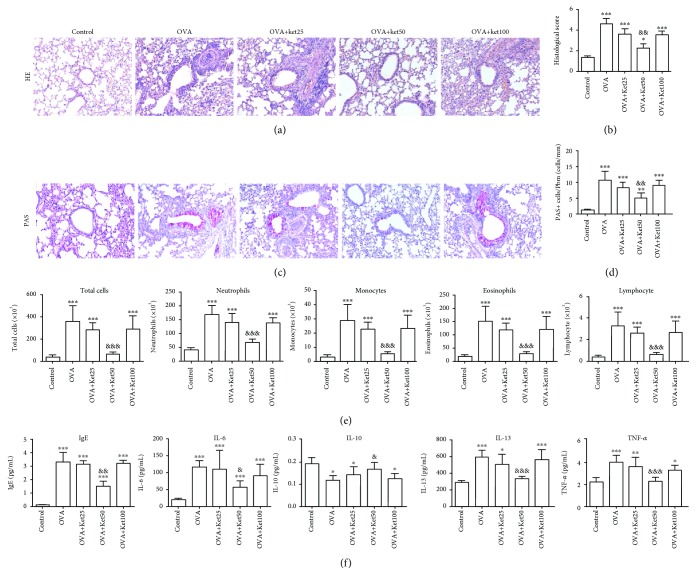
Effect of ketamine on the histological findings, inflammatory cell counts, and cytokine levels in BALF from mice sensitized to OVA. (a, b) H&E-stained lung histology (magnification, ×200) and histopathological score. (c, d) PAS-stained lung histology (magnification, ×200) and quantification of PAS-positive cells. (e) Inflammatory cell counts in BALF. (f) Cytokine levels in BALF. Data are expressed as the mean ± SD of six mice per group. ^∗^*P* < 0.05, ^∗∗^*P* < 0.01, and ^∗∗∗^*P* < 0.001 versus the control group; ^&^*P* < 0.05, ^&&^*P* < 0.01, and ^&&&^*P* < 0.001 versus the OVA group.

To confirm the roles of ketamine in airway inflammation in an OVA-induced allergic model in mice, 25, 50, and 100 mg/kg ketamine were administered to mice i.p. for 7 days. Histological examination showed that the airways from the mice sensitized with OVA were extensively damaged, as indicated by infiltrated inflammatory cells in the subepithelial space (Figure 3(a)). This infiltration was slightly reduced but without statistical difference in the mice exposed to 25 mg/kg ketamine (Figure 3(b)). When the dose level of ketamine was increased to 50 mg/kg, the inflammatory cell infiltration was further suppressed, and statistical significance was generated when compared with the mice challenged with OVA only. Unexpectedly, the amelioration of inflammatory cell infiltration was reversed in the mice treated with 100 mg/kg ketamine. Consistently, the inflammation sore in lung tissues was revealed to have significant reduction in the mice in the OVA+Ket50 group, whereas there was comparable inflammation in the OVA+Ket25/Ket100 group compared with the mice in the OVA group (Figure 3(b)). PAS staining revealed significantly decreased PAS-positive mucous-containing goblet cells in the mice in the OVA+Ket50 group, rather than in the OVA+Ket25/50 group (Figures 3(c) and 3(d)).

Cell counting with BALF showed that the number of total cells was significantly increased in the OVA group (Figure 3(e)). Treatment with 25 or 100 mg/kg ketamine slightly decreased the number but without statistical significance. In mice receiving 50 mg/kg ketamine, however, the cell count was significantly decreased as compared to that of the control. Consistent alterations in the inflammatory cell subtypes, including neutrophils, monocytes, eosinophils, and lymphocytes, were observed in ketamine-treated groups, as compared with the control or OVA groups.

Determination of inflammatory mediators showed that OVA significantly increased the levels of proinflammatory mediators, including IgE, IL-6, IL-13, and TNF-*α*, whereas the levels of the anti-inflammatory cytokine, IL-10, were decreased (Figure 3(f)). After treatment with 50 mg/kg ketamine, alterations induced by OVA were significantly inhibited. However, this inhibitory effect was not observed in mice treated with 25 or 100 mg/kg ketamine.

All of the results mentioned above indicated that allergic inflammation in the airway was successfully established with OVA treatment in this study. Treatment with ketamine at 50 mg/kg significantly inhibited the inflammation in lungs and protected airways induced by OVA.

### 3.2. Roles of Ketamine in OVA-Induced Autophagy

**Figure 4:**
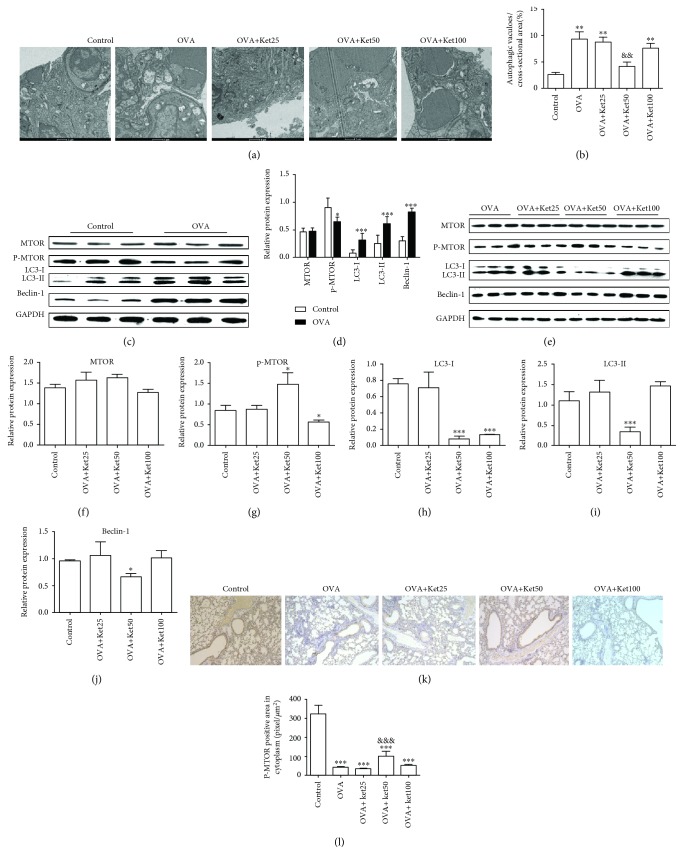
Effect of ketamine on autophagy in the lungs from mice sensitized to OVA. (a, b) Electron micrographs and quantification of autophagic vacuoles. (c–j) With Western blot, MTOR, p-MTOR, LC-3I, LC-3II, and Beclin-1 were determined and quantified. (k, l) With immunohistochemistry (magnification, ×200), p-MTOR were determined and quantified. Data are expressed as the mean ± SD of six mice per group. ^∗^*P* < 0.05, ^∗∗^ *P* < 0.01, and ^∗∗∗^*P* < 0.001 versus the control group; ^&&^*P* < 0.01 and ^&&&^*P* < 0.001 versus the OVA group.

To assess the mechanism by which ketamine exerted a protective effect on OVA-induced asthma, features of autophagy were examined with electron microscopy. As shown in Figure 4(a), double-membrane autophagosomes were more prominent in airways in OVA-challenged mice except for animals treated with 50 mg/kg ketamine, when compared with those in the control. Quantification of total vacuoles on tissue sections also revealed that the number and the area occupied by autophagic vacuoles were greater in the OVA model group than in the control (Figure 4(b)). The enhancement was significantly inhibited by 50 mg/kg ketamine, whereas it was not affected by 25 or 100 mg/kg ketamine. These results indicated that 50 mg/kg ketamine could significantly inhibit autophagy in airways induced by OVA, whereas the effect was not observed after treatment with 25 or 100 mg/kg ketamine.

Consistently, Western blot analysis of the critical proteins in autophagy pathway showed that LC-I, LC-II, and Beclin-1 were significantly increased by OVA treatment (Figures 4(c) and 4(d)). Additionally, OVA treatment significantly decreased the phosphorylation of MTOR. When compared with animals in the OVA model group, significantly increased p-MTOR and decreased LC-I, LC-II, and Beclin-1 in the OVA+KET50 group were observed, while comparable levels of these proteins were seen in OVA+Ket25 and OVA+Ket100, even though significantly decreased p-MOTR and LC-I levels were observed in OVA+Ket100 (Figures 4(e)–4(j)).

In agreement, the level of p-MTOR determined by Western blot and IHC analysis showed that p-MTOR was significantly decreased by OVA treatment, and the inhibitory effect was significantly attenuated by 50 mg/kg ketamine but not by 25 or 100 mg/kg ketamine (Figures 4(k) and 4(l)).

All of the above-mentioned results indicated that OVA treatment significantly induced autophagy in airways in mice. Moreover, this induction was significantly inhibited by 50 mg/kg ketamine, which might be via mitogenic activation of MTOR. However, the effects on autophagy and MTOR activation were not observed after 100 mg/kg ketamine treatment.

### 3.3. Rapamycin Abrogates the Protective Role of 50 mg/kg Ketamine in Allergic Airway Inflammation

**Figure 5:**
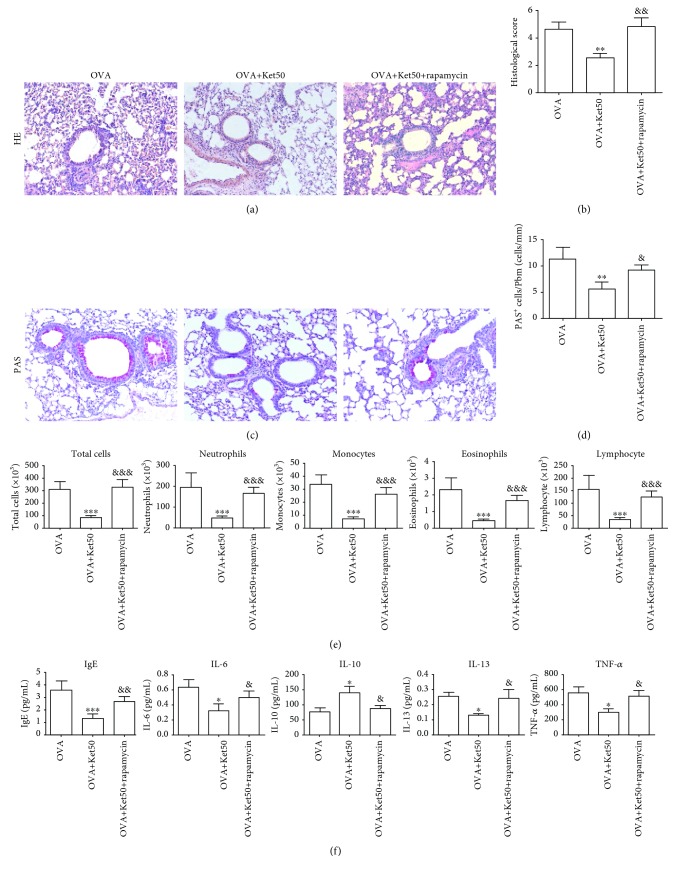
Effect of rapamycin on the histological findings, inflammatory cell counts, and cytokine levels in BALF from asthmatic mice treated with 50 mg/kg ketamine. (a, b) H&E-stained lung histology (magnification, ×200) and histopathological score. (c, d) PAS-stained lung histology (magnification, ×200) and quantification of PAS-positive cells. (e) Inflammatory cell counts in BALF. (f) Cytokine levels in BALF. Data are expressed as the mean ± SD of six mice per group. ^∗^*P* < 0.05, ^∗∗^*P* < 0.01, and ^∗∗∗^*P* < 0.001 versus the OVA groups; ^&^*P* < 0.05, ^&&^*P* < 0.01, and ^&&&^*P* < 0.001 versus the OVA+Ket50group.

OVA-challenged mice were treated with 50 mg/kg ketamine alone or in combination with rapamycin to evaluate whether MTOR played a role in a ketamine-mediated protective effect on asthma. Histological analysis showed that the inhibitory role of 50 mg/kg ketamine in inflammation induced by OVA was blocked by rapamycin treatment (Figures 5(a) and 5(b)). Additionally, rapamycin treatment restored the decreased number of PAS+ mucous-containing goblet cells by 50 mg/kg ketamine, as shown in Figures 5(c) and 5(d). Consistent with the results observed histologically, cell counting showed restored effect by rapamycin on the decreased number of total cells and inflammatory cell subtypes (Figure 5(e)), as well as on the decreased proinflammatory mediators by OVA (Figure 5(f)). Additionally, the enhanced level of IL-10 was returned after treatment with rapamycin. All of these indicated that the protective role of 50 mg/kg ketamine in allergic airway inflammation was significantly inhibited by rapamycin.

### 3.4. Effect of Rapamycin on 50 mg/kg Ketamine-Inhibited Autophagy

**Figure 6:**
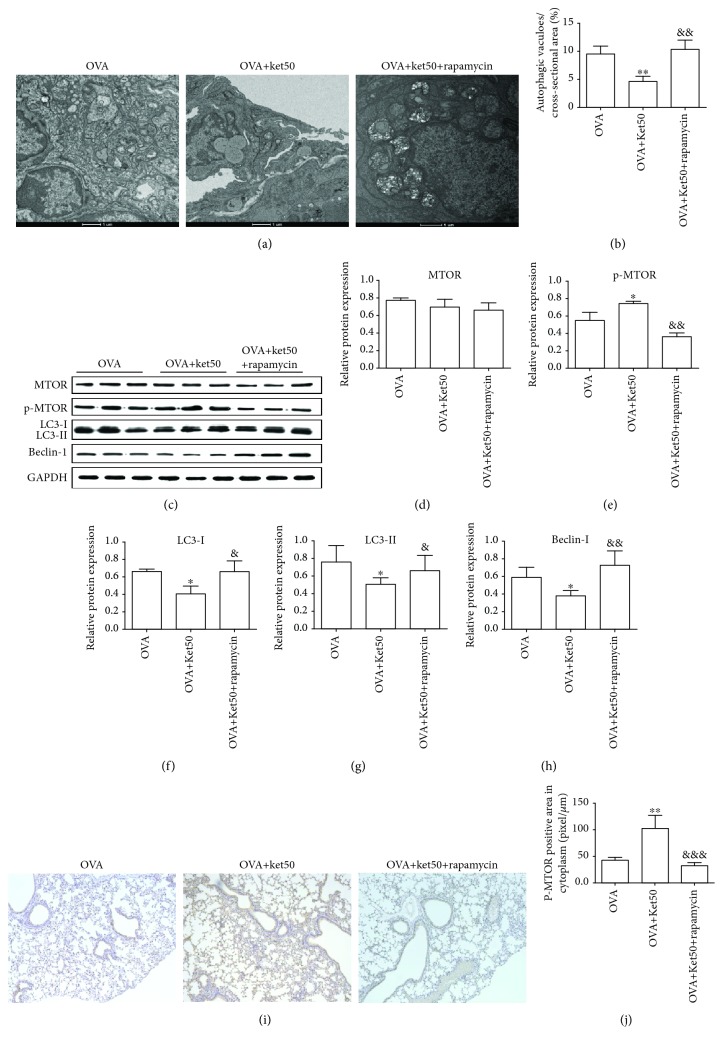
Effect of rapamycin on autophagy in the lungs from asthmatic mice treated with 50 mg/kg ketamine. (a, b) Electron micrographs and quantification of autophagic vacuoles. (c–h) With Western blot, MTOR, p-MTOR, LC-3I, LC-3II, and Beclin-1 were determined and quantified. (i, j) With immunohistochemistry (magnification, ×200), p-MTOR were determined and quantified. Data are expressed as the mean ± SD of six mice per group. ^∗^*P* < 0.05, ^∗∗^*P* < 0.01 versus the OVA groups; ^&^*P* < 0.05, ^&&^*P* < 0.01, and ^&&&^*P* < 0.001 versus the OVA+Ket50 group.

Treatment with rapamycin significantly reversed the inhibited autophagic vacuoles induced by 50 mg/kg ketamine (Figures 6(a) and 6(b)). Western blot assay showed that the significant increase in p-MTOR and decrease in LC-3II and Beclin-1 were rescued by rapamycin treatment (Figures 6(c)–6(h)). Consistent inhibitory effects by rapamycin on the 50 mg/kg ketamine-induced p-MTOR were observed in the IHC analysis (Figures 6(i) and 6(j)). These results indicated that the inhibitory effect by 50 mg/kg ketamine on autophagy was significantly suppressed by rapamycin, and this might be achieved by inhibiting MTOR activation.

## 4. Discussion

Allergic airway inflammation is one of the most common chronic disorders in children, and it is characterized by complex inflammatory responses [17]. Although allergic airway inflammation has been widely researched and therapies have been developed, many patients, especially for those with acute or severe asthma, fail to respond to conventional treatment. For this subpopulation of patients, ketamine has been suggested as a potentially promising therapy [18]. In the present study, the effective response induced by ketamine was observed in a mouse model sensitized to OVA. Furthermore, we for the first time revealed that MTOR activation-induced inhibition of autophagy critically contributed to the effect of ketamine on allergic mice.

Inflammation is a key factor in the initiation and development of allergic airway inflammation, especially allergic asthma [19]. In this study, allergic airway inflammation was successfully established as demonstrated by a significant increase of inflammatory cells and mediators in BALF, including neutrophils, monocytes, eosinophils, and lymphocytes, as well as mediators, such as IgE, IL-6, IL-13, and TNF-*α*. Allergic inflammation was significantly inhibited along with decreased autophagy, and this was rescued by treatment with rapamycin, a specific inhibitor of MTOR, indicating that autophagy might be importantly associated with inflammation. A consistent observation was noted in a recent study with the same mouse model, in which autophagy inhibition by 3-methyladenine or *ATG5* shRNA treatment reduced airway responsiveness, eosinophilia, and inflammation [20]. Another study also indicated that elevated autophagy was essential for airway inflammation induced by particulate matter [21].

Contrary to the finding in this study that MTOR activation contributed to anti-inflammation, a previous study reported that MTOR inhibition decreased inflammatory cell counts, IgE, and IL-13, resulting in anti-inflammation [22]. Pharmacological inhibition or genetic knockdown of MTOR in bone marrow-derived macrophages led to amplified cytokine production upon exposure to particulate matter [9]. The opposite role in inflammation by MTOR inhibition might be due to the variable effects of autophagy on inflammation. As a result of autophagy inhibition, ketamine significantly attenuated allergic airway inflammation in mice, which is similar to previous studies [12]. However, the protective role of autophagy in inflammation has been reported in a recent study, in which mice were sensitized to house dust mite [23]. Another study showed similar data that airway inflammation and remodeling were attenuated by the upregulation of autophagy in mouse models of asthma [24]. The different roles of autophagy mentioned above might be due to different levels of autophagy being provoked by various sensitization methods. Autophagy is believed to be a double-edged sword as both excessive autophagy and impaired autophagy are associated with many diseases [25]. Although the mechanism underlying the difference needs to be elucidated, the pronounced autophagy in severe asthma in mice in the present study was consistent with clinical observations. Furthermore, attenuation of asthma by ketamine in clinics was also observed in this study in mice. This suggests that the new findings in this study might be more consistent with those observed in clinical settings.

However, the inhibitory effect on allergic inflammation by ketamine was reversed at higher dosages. This might be due to the interesting properties of this drug acting as an immunomodulatory agent rather than as an immunosuppressive agent [26]. The reversion was accompanied by decreased MTOR activation and promotion of autophagy. The variable effect on inflammation might be derived from the different roles in MTOR activation and the resultant autophagy.

Electron microscopy in a previous study has shown more autophagosomes in bronchial biopsy tissue from asthmatic patients than from healthy subjects [27]. A study by Pawankar et al. showed that more double-membrane autophagosomes were observed in severe allergic asthma mice [19]. Our study also observed significant induction of autophagy in pulmonary tissues in allergic mice. Furthermore, it was noted that the inhibition of autophagy by ketamine significantly attenuated allergic inflammation, which was rescued by treatment with rapamycin. All of these indicated that autophagy might be a therapeutic target against asthma.

The inhibition of autophagy by ketamine was majorly attributed to the activation of MTOR, which was consistent with a previous study [16]. Unexpectedly, we observed that no changes in p-MTOR, autophagy marker, or autophagosome formation occurred in the higher dosage of ketamine, as compared with the OVA group. Similar to the varied roles in autophagy in asthma, ketamine has been reported to activate or inhibit autophagy, exerting neuroprotection [16, 28]. It has been shown that ketamine at higher concentrations might cause ROS generation, which induces autophagy by MTOR inhibition [29–31]. Additionally, this variable effect on autophagy by different dosages of ketamine might be due to MTOR-mediated inhibition autophagy, and it is balanced by the activation of other pathways that also influence autophagy such as the IP3-Akt pathway, which was demonstrated to be influenced by drugs such as lithium [32]. Regardless of the associated mechanism, the dosage of ketamine should be tightly controlled during treatment of severe asthma, because administration of ketamine at higher concentrations might show negative effects. Accompanied by different effects on autophagy by ketamine, its role in inflammation is varied. However, these effects were blocked following rapamycin treatment, indicating that ketamine plays roles in inflammation through the regulation of autophagy, which subsequently modulates the asthma disease state.

In summary, a mouse model of asthma was successfully established with OVA challenge, and administration of ketamine at a proper dosage induced MTOR phosphorylation, inhibited autophagy, suppressed inflammation, and attenuated OVA-induced asthma. Of note, higher doses of ketamine abolished these effects while lower doses were not effective, which highlights the need for establishing a tight dose range for clinical applications. Although ketamine has been shown to protect against allergic airway inflammation, it is not the first choice for the treatment of allergic airway inflammation in humans. The literature on the potential harm of ketamine abuse is well documented. It has a negative impact on cognitive function including verbal memory and visual recognition difficulties [33]. It is also linked to an increase in the incidence of mental illness such as depression and psychosis [34, 35]. The standard treatment of acute exacerbation of allergic airway disease includes O_2_ supplementation, inhaled beta2-agonist, and systemic corticosteroids [36].

## 5. Conclusions

Taken together, the results of the present study showed that ketamine intraperitoneal injection inhibited the inflammatory cascade response in an experimental allergic airway inflammation model. Ketamine at 50 mg/kg inhibited allergic airway inflammation by suppressing autophagy, and its effect mediated the activation of MTOR. These findings collectively indicate that ketamine might provide a new therapeutic approach for the treatment of allergic asthma.

## Data Availability

The data used to support the findings of this study are included within the article.

## Abbreviations

BALF: Bronchoalveolar lavage fluid
p-MTOR: Phosphorylated mammalian target of rapamycin
HRP: Horseradish peroxidase
IgE: Immunoglobulin E
IL: Interleukin
OVA: Ovalbumin
PAS: Periodic acid-Schiff
Ket: Ketamine
TNF-*α*: Tumor necrosis factor-*α*
DMSO: Dimethyl sulfoxide
ELISA: Enzyme-linked immunosorbent assay
HE: Hematoxylin and eosin.
